# Impaired **β** Cell Function in Chinese Newly Diagnosed Type 2 Diabetes Mellitus with Hyperlipidemia

**DOI:** 10.1155/2014/493039

**Published:** 2014-04-14

**Authors:** Yuhang Ma, Yufan Wang, Qianfang Huang, Qian Ren, Su Chen, Aifang Zhang, Li Zhao, Qin Zhen, Yongde Peng

**Affiliations:** Department of Endocrinology and Metabolism, Shanghai First People's Hospital, Shanghai Jiao Tong University, 100 Haining Road, Shanghai 200080, China

## Abstract

The objective is to explore the effects of hyperlipidemia on **β** cell function in newly diagnosed type 2 diabetes mellitus (T2DM). 208 patients were enrolled in the study and were divided into newly diagnosed T2DM with hyperlipidemia (132 patients) and without hyperlipidemia (76 patients). Demographic data, glucose levels, insulin levels, lipid profiles, homeostasis model assessment for **β** cell function index (HOMA-**β**), homeostasis model assessment for insulin resistance index (HOMA-IR), and quantitative insulin-sensitivity check index (QUICKI) were compared between the two groups. We found that comparing with those of normal lipid levels, the subjects of newly diagnosed T2DM with hyperlipidemia were younger, and had declined HOMA-**β**. However, the levels of HOMA-**β** were comparable regardless of different lipid profiles (combined hyperlipidemia, hypertriglyceridemia, and hypercholesterolemia). Multiple stepwise linear regression analysis showed that high fasting plasma glucose (FPG), decreased fasting insulin level (FINS), and high triglyceride (TG) were independent risk factors of **β** cell dysfunction in newly diagnosed T2DM. Therefore, the management of dyslipidemia, together with glucose control, may be beneficial for T2DM with hyperlipidemia.

## 1. Introduction

Type 2 diabetes mellitus (T2DM) is a chronic metabolic disease with insulin resistance and insulin secretion deficiency being the two major pathophysiological defects. The prevalence of T2DM and prediabetes in China was 9.7% and 15.5%, respectively, which implies that there were 92.4 million adults with diabetes and 148.2 million adults with prediabetes [1]. Hyperlipidemia, one of the most common T2DM related comorbidities, refers to the increase of total cholesterol or/and triglycerides in the serum [2]. On the one hand, insulin resistance diverts carbohydrate away from muscle glycogen storage into hepatic de novo lipogenesis, thus leading to the increase of plasma triglyceride concentration [3]. On the other hand, high-fat diet downregulates hormone-sensitive lipase activity, which promotes diacylglycerol accumulation and lipotoxicity and thus impairs muscular insulin signaling [4].

Lipotoxicity can not only induce insulin resistance but impair *β* cell function as well. Our team previously found that 3T3L1 adipocytes disturbed rat islets insulin secretion in coculture system (the 3T3L1 adipocytes and the rat islet cells). The effects may be mediated by multiple pathways, including the downregulation of glucose-stimulated insulin secretion (GSIS) gene expression, the suppression of islet cell insulin signaling, and the induction of oxidative stress [5]. In vivo, it also suggested that the impaired insulin secretion was accompanied by insulin resistance in the high-fat diet rats [6]. Lipotoxicity to the extent can be attributable to hyperlipidemia [7]. Therefore, we hypothesize that *β* cell function declines in newly diagnosed T2DM with hyperlipidemia comparing their normal lipid profile counterparts.

In this study, we examined the demographic data, glucose levels, insulin levels, lipid profiles, homeostasis model assessment for *β* cell function index (HOMA-*β*), homeostasis model assessment for insulin resistance index (HOMA-IR), and quantitative insulin-sensitivity check index (QUICKI) in newly diagnosed T2DM with hyperlipidemia and without hyperlipidemia.

## 2. Materials and Methods

### 2.1. Subjects

208 newly diagnosed T2DM were enrolled in the study between April 2011 and July 2013. All of them had been diagnosed DM within six months with euthyroidism. Diagnosis of T2DM was based on WHO diagnostic criteria in 1999 [8]. Then, the subjects were divided into two groups according to their serum lipids level: newly diagnosed T2DM with hyperlipidemia and without hyperlipidemia. Hyperlipidemia was defined as serum cholesterol (TCH) was over 5.2 mmol/L or/and serum triglyceride (TG) was over 1.7 mmol/L. Those with hyperlipidemia were subgrouped into combined hyperlipidemia, hypertriglyceridemia, and hypercholesterolemia. Combined hyperlipidemia was defined as both serum TCH was over 5.2 mmol/L and serum TG was over 1.7 mmol/L; hypertriglyceridemia was defined as serum TG was over 1.7 mmol/L; hypercholesterolemia was defined as serum TCH was over 5.2 mmol/L. Each patient was given informed consent; the research was carried out in compliance with the declaration of Helsinki, and the protocol was approved by the ethical committees of Shanghai First People's Hospital, Shanghai Jiao Tong University.

### 2.2. Data Collection

The demographic data and clinical data were collected including age, sex, diabetic duration, body mass index (BMI), TCH level, TG level, high density lipoprotein-cholesterol (HDL-C) level, low density lipoprotein-cholesterol (LDL-C) level, fasting plasma glucose (FPG) level, 2 h postprandial glucose (2hPG) level, fasting insulin (FINS), 2 h postprandial insulin (2hINS), and hemoglobin A1c (HbA1c) level.

HOMA-*β*, HOMA-IR, and QUICKI were calculated to evaluate the *β* cell function, insulin resistance, and insulin sensitivity, respectively. HOMA-*β* = 100 × FINS (*μ*U/mL)/(FPG (mmol/L) −3.5), HOMA-IR = FPG (mmol/L) × FINS (*μ*U/mL)/22.5, and QUICKI = 1/(log (FPG mg/dL)) + log (FINS *μ*U/mL)) [9, 10].

### 2.3. Statistical Analysis

Data were tested for a normal distribution applying the one-sample Kolmogorov-Smirnov test. Variables with skewed distribution (HOMA-*β*) were ln-transformed. Data were shown as means ± SD. *t*-test was used in comparison with the data between two groups. One-way ANOVA test was used for the comparison among the different types of hyperlipidemia. Multiple stepwise linear regression analysis was performed for identification of the risk factors for HOMA-*β*. In multiple stepwise linear regression, a criterion for entry was *P* < 0.05 and for removal was *P* > 0.1. All analyses were performed using SPSS software program (version 17.0), and *P* < 0.05 was considered statistically significant.

## 3. Results 

### 3.1. The Comparison of the Demographic and Clinical Data between Subjects of Newly Diagnosed T2DM with Hyperlipidemia and without Hyperlipidemia

132 (63.5%) T2DM patients had been identified with hyperlipidemia. The male/female ratio had no difference between the two groups (57.6% of male for T2DM with hyperlipidemia and 57.9% of male for those without hyperlipidemia). Subjects of newly diagnosed T2DM with hyperlipidemia were younger (53.41 ± 11.97 years old versus 57.10 ± 11.77 years old, *P* < 0.05), had higher TG level (2.38 ± 1.30 mmol/L versus 1.21 ± 0.29 mmol/L, *P* < 0.01), had higher TCH level (5.50 ± 1.08 mmol/L versus 4.26 ± 0.61 mmol/L, *P* < 0.05), and had higher LDL-C level (3.19 ± 1.08 mmol/L versus 2.57 ± 0.70 mmol/L, *P* < 0.01) compared with those with normal lipids. However, there were no significant differences in BMI, diabetic duration, HDL-C, FPG, 2hPG, FINS, 2hINS, and HbA1c between the two groups (Table 1).

### 3.2. The Comparison of the HOMA-*β*, HOMA-IR, and QUICKI Levels between Subjects of Newly Diagnosed T2DM with Hyperlipidemia and without Hyperlipidemia

**Figure 1:**
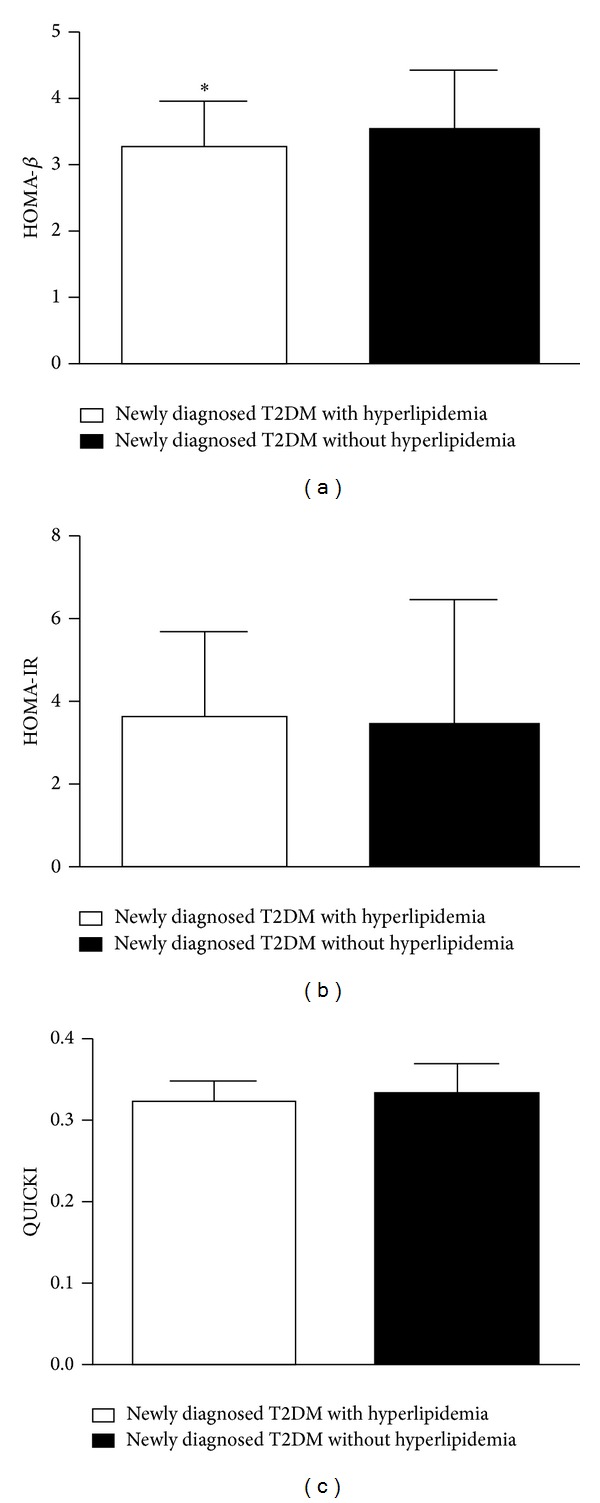
(a) HOMA-*β* level in subjects of newly diagnosed type 2 diabetes mellitus (T2DM) with hyperlipidemia and without hyperlipidemia. (b) HOMA-IR level in subjects of the two groups. (c) QUICKI level in subjects of the two groups. HOMA-*β* was ln-transformed. Hyperlipidemia was defined as serum cholesterol was over 5.2 mmol/L or/and serum triglyceride was over 1.7 mmol/L. *P < 0.05 versus newly diagnosed T2DM without hyperlipidemia.

Compared with the subjects with normal lipids, those of newly diagnosed T2DM with hyperlipidemia have declined HOMA-*β* (hyperlipidemia, 3.28 ± 0.70 versus normal lipids, 3.51 ± 0.90, *P* < 0.05, HOMA-*β* level was ln-transformed) (Figure 1(a)). However, HOMA-IR and QUICKI showed no differences between the two groups (Figures 1(b) and 1(c)).

### 3.3. The Comparison of HOMA-*β* Level in Different Types of Hyperlipidemia in Newly Diagnosed T2DM

**Figure 2:**
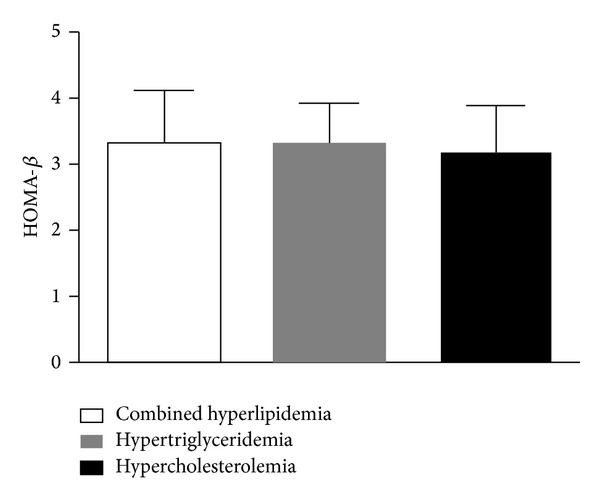
HOMA-*β* levels of subjects of different types of hyperlipidemia in newly diagnosed type 2 diabetes (T2DM). HOMA-*β* had been ln-transformed. Combined hyperlipidemia was defined as serum cholesterol level was over 5.2 mmol/L and serum triglyceride was over 1.7 mmol/L; hypertriglyceridemia was defined as serum triglyceride was over 1.7 mmol/L; hypercholesterolemia was defined as serum cholesterol was over 5.2 mmol/L. The different types of hyperlipidemia seemed to have comparable effect on HOMA-*β* level in newly diagnosed T2DM.

The HOMA-*β* levels were 3.34 ± 0.76, 3.31 ± 0.62, and 3.15 ± 0.73 for subjects with combined hyperlipidemia (*n* = 49, 37.1%), with hypertriglyceridemia (*n* = 50, 37.9%), and with hypercholesterolemia (*n* = 33, 25%), respectively (HOMA-*β* level was ln-transformed). The different types of lipid profiles seemed to have comparable effects on beta cell function in newly diagnosed T2DM (Figure 2).

### 3.4. Multiple Stepwise Linear Regression Analysis

Multiple stepwise linear regression analysis with age, sex, BMI, FINS, 2hINS, FPG, 2hPG, HDL-C, LDL-C, TG, TCH, and HbA1C as independent variables and with HOMA-*β* being dependent variable showed that FPG (standardized coefficient (*β*): −0.497, *P* = 0), FINS (*β*: 0.667, *P* = 0), and TG (*β*: −0.102, *P* = 0.029) were significantly associated with HOMA-*β* of newly diagnosed T2DM.

## 4. Discussion 

T2DM patients have high risks of lipid disorders characterized mainly by elevated levels of TG and LDL-cholesterol [11]. Our data demonstrated that compared with those with the normal lipid levels, newly diagnosed T2DM with hyperlipidemia were more susceptible to impaired *β* cell function. The association between lipid level and *β* cell function has been examined in the previous studies, but most of the evidences were from animal studies. Hao et al. found that apolipoprotein E- (apoE-) deficient mice with standard diet displayed elevated plasma cholesterol levels with plasma free fatty acid unchanged. The elevated serum cholesterol led to increased islet cholesterol and reduced insulin secretion. Moreover, *β* cell could restore normal secretion by cholesterol depletion [12]. Brunham et al. investigated that elevated serum or islet cholesterol could make *β* cell dysfunction and loss of insulin secretion in mice regulated by ATP-binding cassette transporter subfamily A member 1 (ABCA1), a cellular cholesterol transporter [13]. *β* cell specific ABCA1 knockout mice exhibited accumulation of cellular cholesterol, marked reduction in insulin secretion, and significantly impaired glucose tolerance, but insulin sensitivity was unaltered, suggesting a defect in islet function [14]. Hermans et al. indicated that T2DM had a high loss rate of insulin secretion and *β* cell function when they had a high ratio of log(TG)/HDL-C, which was used as the evaluation of atherogenic index of plasma. And a lower ratio of log(TG)/HDL-C would be beneficial to glucose control [15]. Our study was mainly focused on the newly diagnosed T2DM (since *β* cell function decreases linearly with diabetic duration) and explored the association between *β* cell dysfunction and the hyperlipidemia including hypertriglyceridemia or/and hypercholesterolemia. Then, the subjects were further grouped into combined hyperlipidemia, hypertriglyceridemia, and hypercholesterolemia. And the results showed that elevated serum TG and TCH had the comparable effect on *β* cell dysfunction in newly diagnosed T2DM.

Except lipotoxicity, glucotoxicity is widely regarded as a key etiology concerning *β* cell dysfunction [16]. Sustained hyperglycaemia damages *β* cell function through several ways such as the increase of oxidative stress, activation of JNK pathway through activated p38 mitogen-activated protein kinase (p38 MAPK) and protein kinase C (PKC), the reduction of the pancreatic and duodenal homeobox factor-1 (PDX-1) function, and the reduction of ERp46 expression [17–19]. United Kingdom prospective diabetes study (UKPDS) discovered that most of the newly diagnosed T2DM had at least 50% loss of *β* cell function, so were the patients in China [20, 21]. While obesity induced insulin resistance was considered to be an important pathogenesis in American and European T2DM patients [22], *β* cell dysfunction was regarded as the main contributor of newly diagnosed T2DM in Asian [23]. In our study there were no differences in FPG, 2hPG, and HbA1c levels between the two groups, and the diabetic duration was comparable which excluded the deterioration of *β* cell function caused by hyperglycemia and diabetic duration.

The results of multiple regression analysis showed that high FPG, decreased FINS, and high TG level were independent risk factors of *β* cell dysfunction in newly diagnosed T2DM. Our result was consistent with prior finding. Imamura et al. have investigated the association of demographic data, hypertension, lipid level, FPG, and adiposity measures with incident diabetes preceding chiefly by insulin resistance, *β* cell dysfunction, or both in an 18-year prospective cohort study. They indicated that higher TG level (≥1.69 mmol/L) and FPG (5.5–7.0 mmol/L) were associated with a higher risk of DM preceded predominantly by *β* cell dysfunction (HR = 1.75, 95% CI 1.04–2.94 and HR = 4.82, 95% CI 2.75–8.46, resp.) [24]. Excess TG caused the elevated levels of circulating free fatty acids (FFAs) by lipolysis and then high concentrations of FFAs impaired *β* cell function [25]. The mechanism included overexpression of G-protein coupled receptor (GPR40) in *β* cells [26], triggering *β* cell apoptosis through the increase of nitric oxide (NO) production and de novo ceramide formation, oxidative stress, and endoplasmic reticulum stress (ER stress) [27–29]. Therefore, the well control of TG level was vital to normal *β* cell function.

Dietary energy restriction (600 kcal/day diet) alone reduced pancreatic and liver triglyceride stores and could recover *β* cell function and hepatic insulin sensitivity in T2DM patients [30]. Antihyperlipidemic agents could improve *β* cell function and delay the need for insulin in T2DM patients as well [31]. A multivariate analysis proved that statins could delay 10 months in the need to commence insulin and cerivastatin could improve first-phase insulin secretion and increase insulin-mediated glucose uptake in the early state of obese T2DM [32]. It emphasized the role of lipid adjustment therapy in both *β* cell protection and glucose control.

## 5. Conclusions 

Newly diagnosed T2DM with hyperlipidemia have impaired *β* cell function compared with those with normal lipid profiles. High FPG, decreased FINS, and high triglyceride are independent risk factors of *β* cell dysfunction for those patients. Therefore, the management of dyslipidemia and hyperglycemia is comparably crucial for T2DM with hyperlipidemia.

## Figures and Tables

**Table 1:** Demographic and clinical data between newly diagnosed T2DM with and without hyperlipidemia.

	Newly diagnosed T2DM with hyperlipidemia	Newly diagnosed T2DM without hyperlipidemia
*N* (M/F)	132 (76/56)	76 (44/32)
Age (years)	53.41 ± 11.97*	57.10 ± 11.77
Diabetic duration (months)	2.09 ± 1.64	2.01 ± 1.38
BMI (kg/m^2^)	25.45 ± 3.25	24.67 ± 3.52
TG (mmol/L)	2.38 ± 1.30**	1.21 ± 0.29
TCH (mmol/L)	5.50 ± 1.08**	4.26 ± 0.61
HDL-C (mmol/L)	1.17 ± 0.28	1.18 ± 0.28
LDL-C (mmol/L)	3.52 ± 1.10**	2.57 ± 0.70
FPG (mmol/L)	9.60 ± 2.73	8.84 ± 3.49
2hPG (mmol/L)	17.20 ± 4.61	16.06 ± 5.64
FINS (pmol/L)	59.41 ± 30.99	60.73 ± 43.10
2hINS (pmol/L)	322.45 ± 325.11	353.96 ± 246.03
HbA1C (%)	9.31 ± 2.27	8.93 ± 2.76

Data are expressed as mean ± SD.

BMI: body mass index; TCH: total cholesterol; TG: triglyceride; HDL-C: high density lipoprotein-cholesterol; LDL-C: low density lipoprotein-cholesterol; FPG: fasting plasma glucose; 2hPG: 2h postprandial glucose; FINS: fasting insulin; 2hINS: 2h postprandial serum insulin; HbA1c: hemoglobin A1c.

Hyperlipidemia was defined as serum cholesterol was over 5.2 mmol/L or/and serum triglyceride was over 1.7 mmol/L. *P < 0.05 versus newly diagnosed T2DM without hyperlipidemia; **P < 0.01 versus newly diagnosed T2DM without hyperlipidemia.

## Abbreviations

BMI: Body mass index
TCH: Total cholesterol
TG: Triglyceride
HDL-C: High density lipoprotein-cholesterol
LDL-C: Low density lipoprotein-cholesterol
FPG: Fasting plasma glucose
2hPG: 2h postprandial glucose
FINS: Fasting insulin
2hINS: 2h postprandial insulin
HbA1c: Hemoglobin A1c
HOMA-IR: Homeostasis model assessment for insulin resistance index
QUICKI: Quantitative insulin-sensitivity check index
HOMA-*β*: Homeostasis model assessment for *β* cell function index.
